# Below-ground herbivory mitigates biomass loss from above-ground herbivory of nitrogen fertilized plants

**DOI:** 10.1038/s41598-020-69696-3

**Published:** 2020-07-29

**Authors:** Pernilla Borgström, Riccardo Bommarco, Maria Viketoft, Joachim Strengbom

**Affiliations:** Department of Ecology, Swedish University of Agricultural Sciences, 75007 Uppsala, Sweden

**Keywords:** Grassland ecology, Ecosystem ecology

## Abstract

Herbivorous insects can influence grassland ecosystem functions in several ways, notably by altering primary production and nutrient turnover. Interactions between above- and belowground herbivory could affect these functions; an effect that might be modified by nitrogen (N) addition, an important global change driver. To explore this, we added above- (grasshoppers) and belowground (wireworms) insect herbivores and N into enclosed, equally composed, grassland plant communities in a fully factorial field experiment. N addition substantially altered the impact of above- and belowground herbivory on ecosystem functioning. Herbivory and N interacted such that biomass was reduced under above ground herbivory and high N input, while plant biomass remained stable under simultaneous above- and belowground herbivory. Aboveground herbivory lowered nutrient turnover rate in the soil, while belowground herbivory mitigated the effect of aboveground herbivory. Soil decomposition potential and N mineralization rate were faster under belowground herbivory at ambient N, but at elevated N this effect was only observed when aboveground herbivores were also present. We found that N addition does not only influence productivity directly (repeatedly shown by others), but also appears to influence productivity by herbivory mediated effects on nutrient dynamics, which highlights the importance of a better understanding of complex biotic interactions.

## Introduction

Nitrogen (N) enrichment is an important driver of global change, as it relaxes N limitation and alters plant community productivity^1^. Increased productivity commonly leads to changed competitive hierarchies among plants, resulting in altered plant species composition and reduced species richness^2,3^. Elevated N input results in increase plant productivity, both directly by increased N availability and indirectly by altering nutrient dynamics^4^. The latter by altered species composition of the plant community^5^, changed plant litter quality^6^, or by changes in function and structure of the soil microbial communities^7,8^. The different directions that ecosystem responses to N addition can take is probably in part due to the mediating role of biotic drivers of the ecosystem, such as herbivory^9,10^. In addition to plant community composition, herbivory can influence ecosystem functions such as nutrient dynamics^5,9,11^, this is particularly true in N-limited environments^12^. Nevertheless, the importance of the interplay between consumer and resource control of ecosystem functioning under N enrichment remains largely unknown.

Herbivorous insects play an important role in determining the rate and direction of many ecosystem processes, with consequences for ecosystem functioning^11,12^. In grasslands, insect herbivory can alter primary productivity^9,13,14^ and nutrient turnover^9,15,16^. A potentially important, but poorly explored aspect of trophic control of grassland ecosystem functioning is that there are herbivores both above and below ground. Although links between above- and belowground trophic interactions are likely to influence ecosystem process rates over time^17^, and have been suggested as being critical for understanding and predicting the ecosystem-level impact of global change^18^, but have so far not been thoroughly explored in controlled experiments.

Insect herbivores can influence ecosystem process rates in several ways^11^. Insect herbivory can accelerate^9,15^ or decelerate^9,19^ nutrient turnover and primary productivity, depending on soil fertility^20^. However, so far most of the knowledge stems from studies of aboveground herbivory. Belowground herbivores are a diverse and abundant group that can have considerable impact on plant communities^21,22^, soil process rates^23^, and feeding behavior and performance of aboveground herbivores^24,25^. Despite this, there is little experimental evidence of the separate and combined effects of above- and belowground herbivory on ecosystem functioning, and even less is known about how such interactions modulate impacts of N addition on the ecosystem.

Although ecosystem-level responses to elevated N input often appears to be context dependent^4^, studies explicitly exploring the role of biotic interactions are still not common. Given the variation in response among different studies and the conclusive evidence that herbivores are key players in determining nutrient turnover and productivity, a crucial next step is to investigate if and how herbivores might mediate the ecosystem’s response to changes in N levels. In this study, we assessed how above- and belowground insect herbivory mediated the response of a grassland ecosystem to N addition. In a field experiment, we set up enclosures containing fertilized or unfertilized grassland plant communities subjected to above- (grasshoppers) and/or belowground (wireworms) insect herbivores. We measured the effects of the treatments on four ecosystem functions: plant biomass production, decomposition rate of a standardized substrate (tea bags), litter decomposability, and N mineralization rate. We tested the general predictions that the overall effect of herbivory on ecosystem functioning would depend on whether both above- and belowground herbivores were present, and that N addition would alter the influence of herbivory.

## Methods

### Experimental design

**Figure 1:**
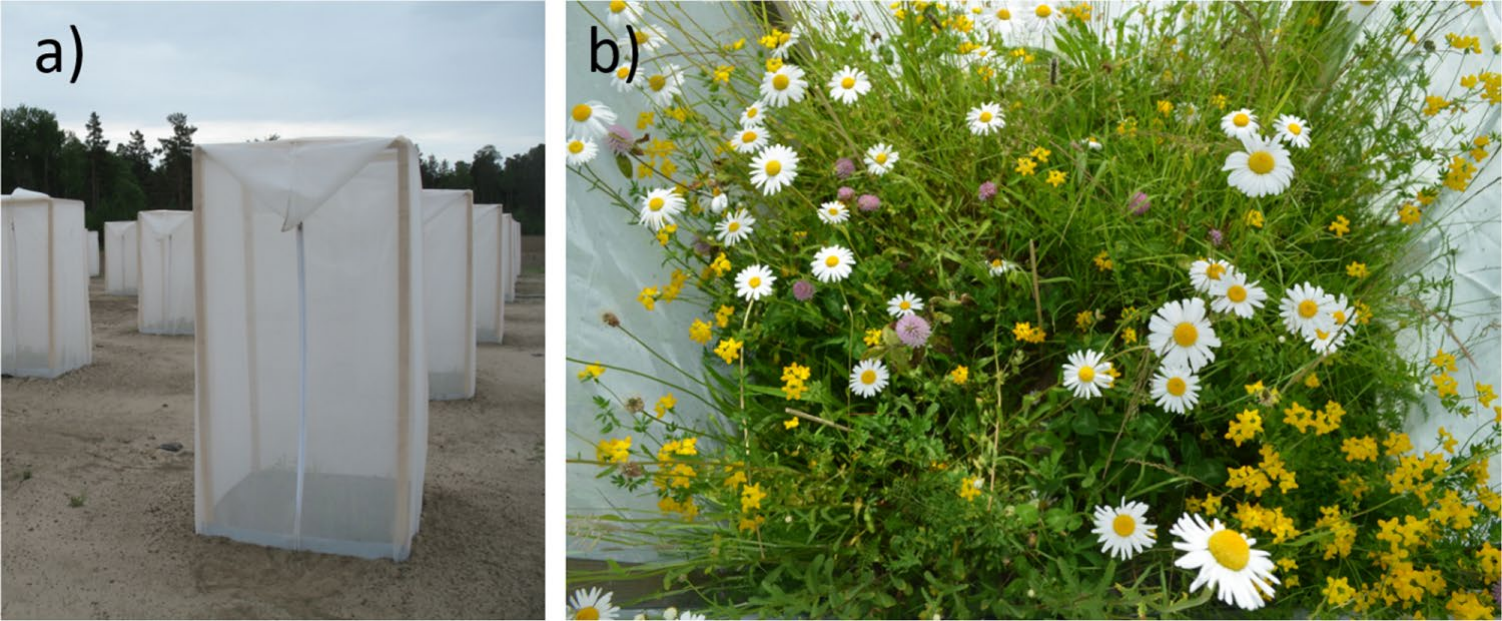
Part of the enclosures (sized 1 × 1 × 2 m) used in the experiment at the initiation of the experiment in 2013 (**a**), and the plat community inside one of the enclosures in 2015 (**b**).

We measured the responses of plant community biomass, nitrogen mineralization rate, soil decomposition potential and litter decomposability, to the three treatments nitrogen (N) addition and above- and belowground insect herbivory in a fully factorial block experiment. Each treatment was replicated once within each block, giving a total of 64 experimental enclosed one by one m plots (Fig. 1). The experiment was established in the spring of 2013 in an organic agricultural field (59° 44′ 27.9″ N, 17° 41′ 02.9″ E) near Uppsala, Sweden, with sandy soil, low soil carbon content and a total N content of 0.08%. In each of the plots, we established plant communities of nine common grassland species: four grasses (*Agrostis capillaris* L., *Dactylis glomerata* L., *Festuca rubra* L., *Lolium perenne* L.), three non-leguminous forbs (*Achillea millefolium* L., *Leucanthemum vulgare* Lam., *Plantago lanceolata* L.), and two leguminous forbs (*Lotus corniculatus* L., *Trifolium pratense* L.). The plant communities were established in late May 2013 from seedlings from seeds (Herbiseed, Twyford, United Kingdom) that were sown in the greenhouse 6–8 weeks prior to planting. All plant communities had the same species composition and all species were planted at equal starting densities in a 1 × 1 m plot of soil. Plants were spaced 10 cm apart in a 9 × 9 grid formation. The position of individual plants within enclosures was randomized for each enclosure, i.e. the planting scheme was not the same in any two enclosures. We manually weeded all enclosures (i.e. removed all plants emerging from the soil’s seed bank) on one occasion during the month that followed planting. In addition, we removed all weeds that we encountered during the subsequent harvests (see “Plant community biomass” section).

The plant communities were enclosed above ground by a mesh net cage (mesh size 0.2 × 0.4 mm, anti-aphid net 20/12, Artes Politecnica, Schio, Italy) of 2 m height, and below ground, by a sheet metal frame of 0.5 m depth with a mesh net bottom. The net had a vertical zipper on one side that allowed entry to the cage. Prior to planting, the base frame was re-filled with soil from the field to a depth of 0.5 m. By refilling the enclosures with unsterilized soil we accepted an uncontrolled level of background herbivory, from insects dwelling in or hatching from of the soil. We considered this natural background herbivory to be preferable to the unrealistic conditions of a fully sterile soil.

### N additions

The N treatment corresponded to 40 kg N ha^−1^ year^−1^, which is in the upper range of current N deposition levels for Western European grasslands^2^, were applied at three occasions,early July 2013, late June 2014, and early July 2015. We applied the N by dissolving ammonium nitrate in 10 l that was watered in the enclosure. Unfertilized enclosures received the same amount of water. No further watering of the plots was carried out.

### Herbivory

The aboveground herbivory treatment consisted of adults of the grasshopper *Chorthippus albomarginatus* (De Geer), added in mid-July 2013 at a density of 10 individuals per enclosure (5 females and 5 males) and allowed to reproduce in the enclosures in subsequent years. We chose the study species as it is one of the most common grasshoppers in the area, and all specimens were collected within a radius of approximately 5 km from the experimental site. For the belowground herbivory treatment, we added wireworms, a common generalist root herbivores in European grasslands, which are the larval stage of the click beetle genus *Agriotes* spp, in mid-July 2013 at a density of 10 individuals per cage. In July 2014, we estimated grasshopper density by counting the individuals in each of the enclosures. Statistical analysis showed that differences in emerged number of nymphs among enclosures were unrelated to N addition (F_1,21_ = 0.095, *p* = 0.76) and belowground herbivory (F_1,21_ = 0.24, *p* = 0.63). Based on the counting, we adjusted grasshopper densities with in each treatment combination. We did this systematically, by dividing the enclosures into quartiles, based on grasshopper density. We did not adjust densities from plots ending up within the two mid-quartiles (19–31 grasshoppers per enclosure), but we transferred grasshoppers from enclosures within the highest quartile to those within the lowest quartile, to give 25 individuals per enclosure. At the time of adjusting the grasshopper densities, we also added 10 extra individuals of the wireworms to each enclosure assigned to this treatment. In 2015, we made no adjustments of herbivore densities. However, we assessed grasshopper densities by visual counting, and grasshopper densities were unaffected by N (F_1,21_ = 3.2; *p* = 0.09) and belowground herbivory (F_1,21_ = 0.7; *p* = 0.4).

### Plant community biomass

Aboveground plant community biomass, henceforth denoted as total shoot biomass, was measured in mid-September 2013, 2014 and 2015, by harvesting the plants. The timing of the harvests corresponded to the peak of standing biomass in the communities. Because all plants renew their aboveground biomass annually, the harvested biomass approximates the annual aboveground production. At harvest, we cut all aboveground plants at 5 cm height above the soil surface. All collected plant material was brought to the lab and oven-dried at 65 °C for 48 h. To simulate the management of a semi-natural grassland, we conducted an additional harvest in mid-June. This harvest was, however, not repeated in 2015 as the plants were left so they could go to seed for a parallel study on plant reproduction.

Belowground plant community biomass, henceforth denoted as total root biomass, was assessed in September 2015 at the end of the 3-year experiment. We collected five soil cores from each enclosure, to a depth of 15 cm, using a cylindrical soil corer (*ϕ*10 cm). We pooled the five cores into one composite sample (volume of 5.9 dm^3^). The samples were kept refrigerated at 4 °C for 3 months before sieving, first with a 5 mm mesh and then a 2 mm mesh sieve. Prior to the second sieving the samples were left to dry for 3 days to facilitate the separation of soil and roots. Finally, the root samples were oven-dried at 65 °C for 48 h, and thereafter weighed.

### Decomposition potential of the soil

To assess decomposition potential of the soil under the different herbivory and nitrogen treatments, we used the tea bag method that produces standardized decomposition rate estimates^26^. We used two types of tea, Lipton Rooibos and Lipton Green Tea (Unilever Belgium, Brussels, Belgium). In in mid-June 2015, we buried (depth of c. 8 cm) two bags of rooibos tea (recalcitrant) and two bags of green tea (easily degradable) in the soil of each enclosure. All tea bags were weighed before being buried. After 90 days, we dug up, dried at 70 °C for 48 h, and re-weighed the bags. The mass loss of the tea bag was used as an estimate of decomposition potential of the soil.

### Decomposition of plant litter

To assess treatment effects on the decomposition of plant litter produced in the enclosures, we used dried plant material from the September 2014 harvest. The decomposition was measured using plant litter harvested from the same enclosure. The material was stored under cool, dry conditions from the harvest in September 2014 until May 2015, when litter for *P. lanceolata*, *T. pratense*, and *D. glomerata* was extracted for litterbag construction. We used these species as they based on the 2014 biomass harvest were dominant species, and as they represent three functional groups, namely grasses (*D. glomerata*), non-leguminous forbs (*P. lanceolata*) and leguminous forbs (*T. pratense*).

The litterbags were manufactured using polyamide mesh (Sefar Nitex 03—50/37, Sintab, Oxie, Sweden) with a pore size of 50 µm, which allows entry of bacteria, fungi and certain microfauna only. Approximately 1 g of dry litter of *P. lanceolata*, *T. pratense*, or *D. glomerata* was enclosed in a right triangular bag with 14 cm sides. After sealing and weighing we placed the bags (one for each plant species), on the soil surface in the enclosure from which the litter originated. We put out the litterbags in mid-June 2015, and collected them after 90 days, when we after dried at 70 °C for 48 h and weighed them. Litter mass loss over the experimental period was then used as an estimate of plant litter decomposability.

### Nitrogen mineralization rate

We assessed the amount of inorganic N mineralized over the growing season in the enclosures with the buried bag technique^26,27^. A soil sample of about 300 g was taken from each enclosure in mid-June 2015 by extracting six evenly spaced cores (*ϕ*25 mm, 10 cm deep) and mixing them into one composite sample. We spitted the composite sample into two samples and put them in polyethylene bags. One bag was buried c. 8 cm below the soil surface in the middle of the enclosure, and the other was brought back to the lab for storage in a freezer (− 20 °C) until analysis. After 90 days, we recollected the buried bags, brought them to the lab and stored them in a freezer until further analyses. These samples were later analyzed for inorganic N content (g/kg of NO_3_/NO_2_ and NH_4_ respectively) using 2 M KCl extraction (Agrilab AB, Uppsala, Sweden). We estimate soil net N mineralization produced over the 90-day period by was subtracting the amount of nitrate and ammonium in the control bags from that in the buried bags.

### Statistical analyses

We used linear mixed effects models^28^ to test how primary production, soil decomposition potential, N mineralization, and litter decomposability responded to N fertilization and above- and belowground herbivory.

Total shoot biomass was analyzed as a dependent variable of the fixed factors N and above- and belowground herbivory, including all possible interactions between the three factors. Year was included as a fixed factor, to account for variation among the harvests in the different years. Enclosure was nested within block in the random structure of the model. To account for autocorrelation of repeated measures within enclosures, we added a first-order autoregressive correlation structure to the model. Total root biomass was analysed with N and above- and belowground herbivory as fixed factors, with all possible interactions among the factors, and block included as a random factor. We also tested for treatment effects on the ratio between root and shoot production in 2015. Finally, we explored whether herbivory generated effects on aboveground shoot biomass were dependent on differences in soil decomposability (mass loss of read tea) and nitrate production by including these variables as covariates in the analyses.

In the analysis of mass loss of the red and green tea, we first calculated the mean mass loss for each tea type (i.e. the mean for the two bags of each type) in each enclosure. The mean mass losses of each tea type and the litter mass losses of *D. glomerata*, *P. lanceolata* and *T. pratense* were then analyzed in linear mixed effects models with block as random factor. In each model, N and above- and belowground herbivory were included as fixed factors, including all possible interactions between the three. For *T. pratense*, two enclosures were excluded as there was no *T. pratense* litter from 2014 to use (i.e. the species had gone extinct in these enclosures). A third enclosure was excluded from the analysis, as its amount of *T. pratense* litter from 2014 was only 0.1 g and skewed the analysis. All analyses were performed in R version 3.2.3 (2015).

## Results

### Total shoot and root biomass

**Figure 2:**
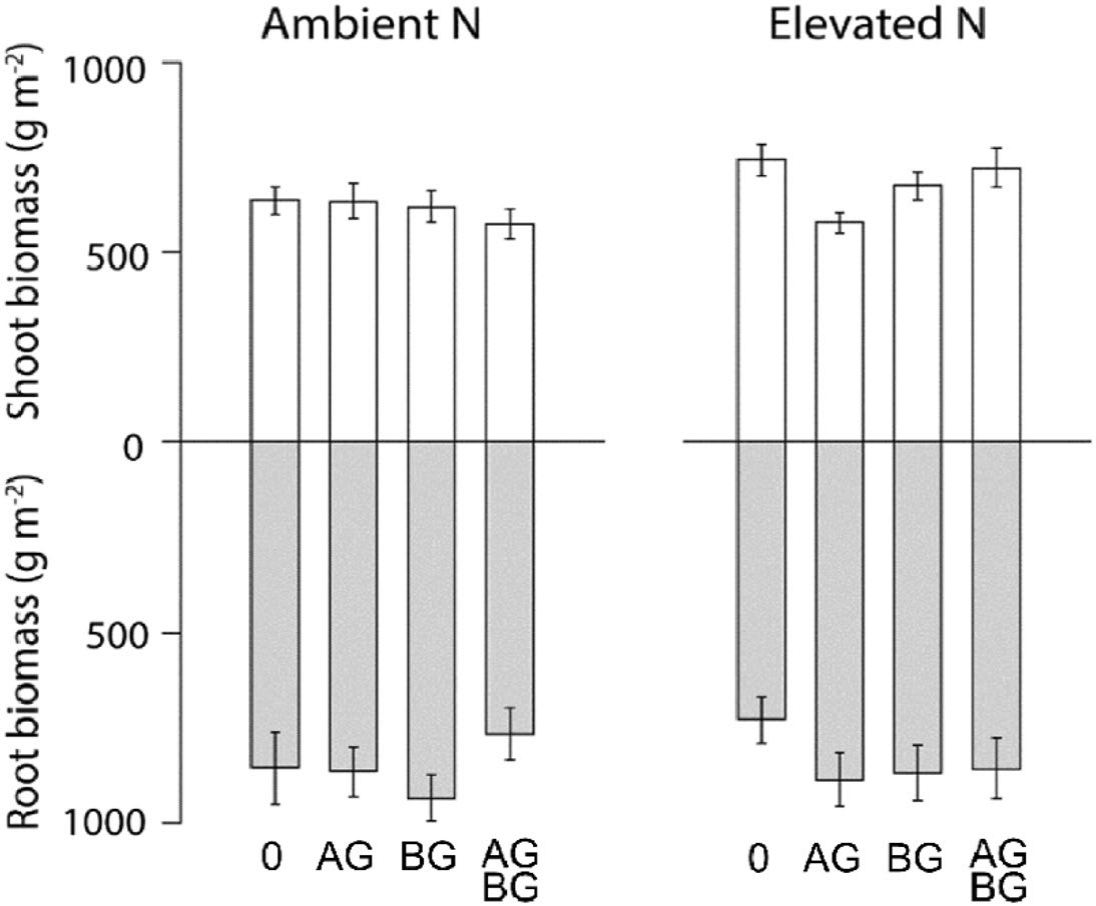
Total root and shoot biomass in the mesocosms in September 2015 at ambient and elevated nitrogen levels. 0 = herbivore-free controls, AG = with aboveground herbivory, BG = with belowground herbivory, AGBG = with above- and belowground herbivory. Height of bars represent treatment averages ± s.e. Note that root biomass is extrapolated from a subsample.

Total shoot biomass was higher at elevated N than at ambient N (Table 1; Fig. 2). This effect was, however, not independent of the effects of above- (A) and belowground (B) herbivory (N × A × B interaction: Table 1a; Fig. 2). At ambient N, above- and belowground herbivory, when applied together, reduced aboveground plant biomass. At elevated N, aboveground herbivory alone reduced plant biomass, while there was no reduction when above- and belowground herbivory were combined (Table 1a; Fig. 1). When mass loss of read tea and nitrate production (see further down) were added as covariates in the analysis the main effect of aboveground herbivory was lost (Supplementary information, Table S2–S3), demonstrating a correlative relationship between these variables.

**Table 1:** Treatment effects of nitrogen (N), aboveground herbivory (A) and belowground herbivory (B) on shoot biomass (aboveground biomass), decomposition rate of red tea, nitrate (NO_3_^−^) production, and litter of *D. glomerata*, *P. lanceolata*, and *T. pratense*.

Treatment	a) Shoot biomass		b) Red tea		c) NO_3_−		d) *D. glomerata*		e) *P. lanceolata*		f) *T. pratense*	
	*F*	*p*	*F*	*p*	*F*	*p*	*F*	*p*	*F*	*p*	*F*	*p*
Year	**344.4**	**< 0.0001**	–	–	–	–	–	–	–	–	–	–
A	**6.9**	**0.01**	0.02	0.9	1.4	0.2	0.2	0.7	3.4	0.07	1.2	0.3
B	0.01	0.9	**4.4**	**0.04**	1.5	0.2	0.2	0.6	1.7	0.2	0.4	0.5
N	**33.3**	**< 0.0001**	0.2	0.6	3.6	0.1	0.2	0.6	1.3	0.3	2.9	0.1
A × B	2.5	0.1	0.01	0.9	0.005	0.9	1.1	0.3	0.1	0.8	2.1	0.2
N × A	0.6	0.4	1.6	0.2	**5.3**	**0.03**	0.6	0.5	0.0	1.0	**7.5**	**0.01**
N × B	3.2	0.1	0.1	0.7	0.4	0.5	0.5	0.5	0.2	0.6	0.9	0.3
N × A × B	**12.0**	**0.001**	**5.4**	**0.02**	**3.9**	**0.05**	**7.3**	**0.01**	2.5	0.1	3.1	0.1

Bold type denotes statistical significance at* p* < 0.05.

Total root biomass was unaffected by all treatments (Fig. 2.). The ratio between root and shoot biomass was higher under aboveground herbivory, but belowground herbivory reversed this effect (A × B interaction; F_1,49_ = 5.5, *p* = 0.03; Fig. 2).

### Decomposition potential of the soil

**Figure 3:**
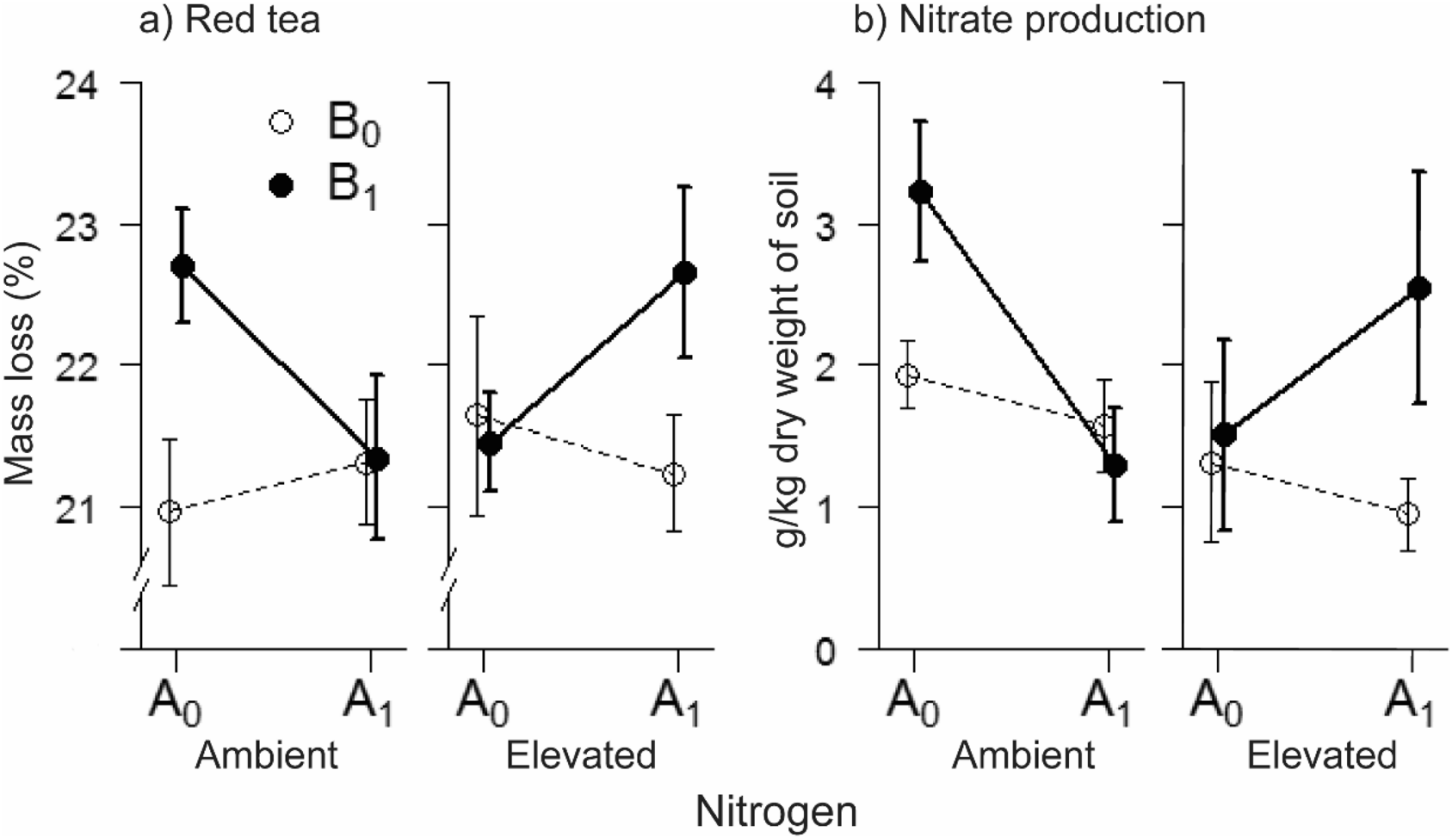
Decomposition potential of the soil, measured as mass loss of red tea (**a**), and nitrogen mineralization, measured as production of nitrate over the growing season (**b**). The plots display treatment averages ± s.e. A0 on the x axis denotes treatments without aboveground herbivory, and A1 treatments with aboveground herbivory. Open circles represent treatments without belowground herbivory (B0), and filled circles treatments with belowground herbivory (B1). N = nitrogen.

Above- and belowground herbivory synergistically influenced the decomposition potential of the soil, as measured by the mass loss of the red tea (N × A × B interaction; Table 1). At ambient N, mass loss was higher when belowground herbivores were present, but only in the absence of aboveground herbivores (Fig. 3a). At elevated N, the interactive effect of above- and belowground herbivory was inverted: presence of belowground herbivores had no influence on mass loss unless aboveground herbivores were also present, in which case mass loss increased (Fig. 3a). Mass loss of the green tea was unaffected by all treatments (*p* > 0.14).

### Nitrogen mineralization

The response of the N mineralization rate to herbivory and N addition mirrored that of the red tea decomposition (compare Fig. 3a, b), with no main effect of N addition but interactive effects with above- and belowground herbivory. Nitrate production dominated the N mineralization and little ammonium was produced. At ambient N, the amount of nitrate produced in the soil was higher in the presence of belowground herbivores. At elevated N, the presence of belowground herbivores only had a positive effect on nitrate production when aboveground herbivores were also present (N × A × B interaction; Table 1).

### Decomposability of plant litter

**Figure 4:**
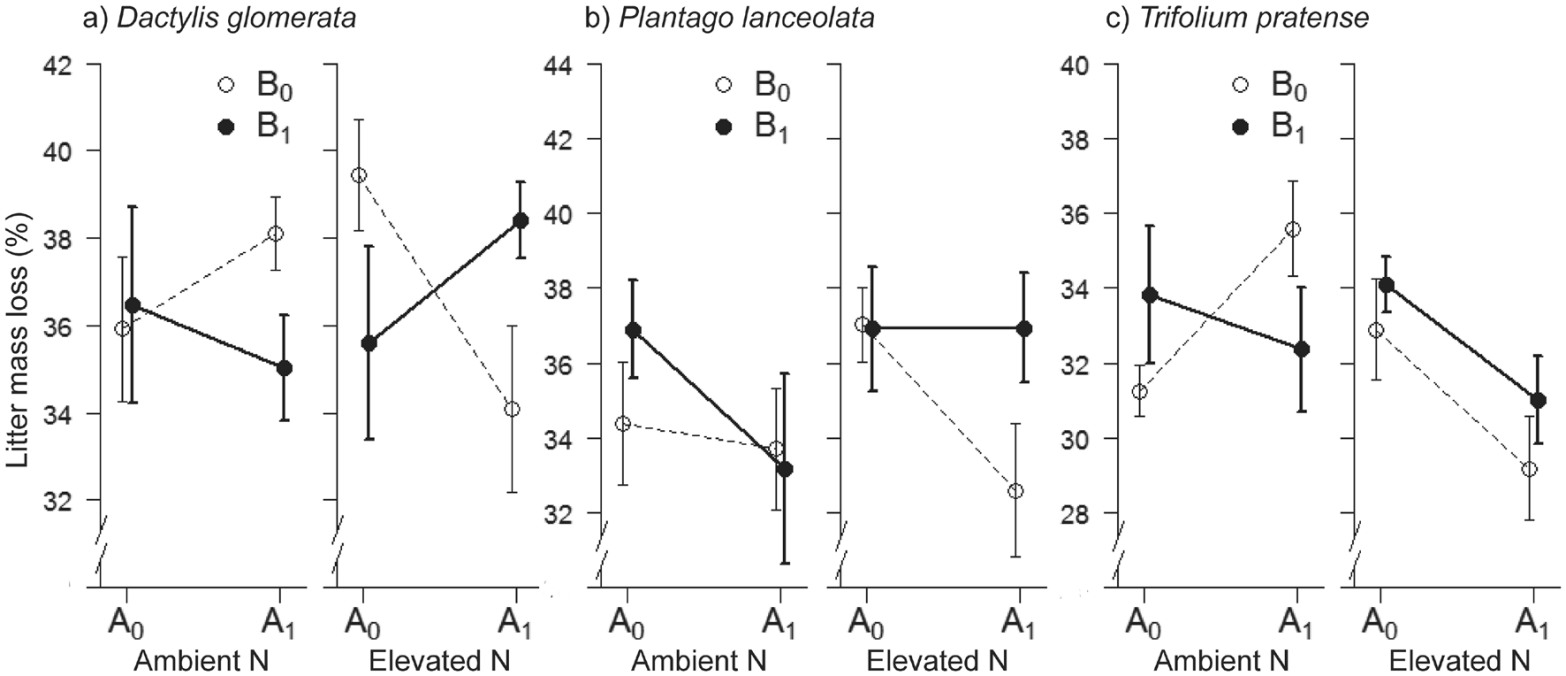
Decomposition of plant litter in relation to above- and belowground herbivory and N enrichment. From left to right showing the response of litter of *D. glomerata* (**a**), *P. lanceolata*, (**b**), and *T. pratense* (**c**). Plant material originated from the same enclosure used for the decomposition experiment. The plots display treatment averages ± s.e. A_0_ on the x axis denotes treatments without aboveground herbivory, and A_1_ treatments with aboveground herbivory. Open circles represent treatments without belowground herbivory (B_0_), and filled circles treatments with belowground herbivory (B_1_). N = nitrogen.

The decomposability of *D. glomerata* litter, expressed as litter mass loss, was synergistically affected by the nitrogen and herbivory treatments (N × A × B interaction; Table 1d; Fig. 4a). Litter mass loss of *D. glomerata* was lower when aboveground herbivores were present, but only at elevated N. The presence of belowground herbivores counteracted the aboveground herbivory effect (Fig. 4a). The decomposition rate of *P. lanceolata* was unaffected by the treatments (Table 1e; Fig. 3b). The decomposition rate of *T. pratense* was lower when aboveground herbivores were present, but only at elevated N (N × A interaction; Table 1f; Fig. 4c). Although there were clear treatment induced effects on the decomposability for two out of three species, the difference in decomposability among species was small (Fig. 4).

## Discussion

We found that the effects of N addition on important ecosystem functions are modulated by, and strongly dependent on, interactions between above- and belowground herbivores. Nitrogen addition has previously been found to accelerate nitrification rates^29^ and soil decomposition processes^4^. We found no increase in these rates from N addition by itself. Rather, it was the presence of herbivory—and specifically, the combination of above- and belowground herbivory—that accelerated the nutrient turnover at elevated N. Aboveground insect herbivory has been shown to both accelerate^15^ and decelerate^30^ nutrient turnover in grasslands. We found striking interactive effects on nutrient turnover related variables (red tea mass loss, litter mass loss of the grass *D. glomerata*, and nitrogen mineralization), which displayed inverted responses to above- and belowground herbivory at contrasting N levels (Figs. 2, 3), suggesting that effects of insect herbivory on nutrient dynamics is strongly context dependent. Such context dependence might help explaining the lack of consensus on how insect herbivory affect nutrient turnover.

There is a great potential for feedbacks between above- and belowground communities and processes. For example, although herbivores via their consumption reduce plant biomass, herbivory can simultaneously stimulate N mineralization, and thereby boost plant productivity^5,15^. We observed higher aboveground biomass production when above- and belowground herbivory were combined, but only at elevated N. We suggest that this is a consequence of the enhanced nutrient turnover caused by combined below- and aboveground herbivory that we also observed (Figs. 1, 2b). Although the mechanism remains unknown, our result indicates that above- and belowground herbivory interactively generate feedbacks on grassland nutrient cycling that determine the response of the plant community to increased N input, and ultimately biomass production.

The effect of herbivores on nutrient turnover can occur by increased root exudations that stimulate microbial growth^31,32^, through alterations in the amount and quality of litter input^33^ or by production of faeces^34^ that all stimulate soil mineralization rates. Although additional measurements are needed to determine the relative importance of these pathways, based on the patterns we observed, it appears as if changes in the litter quantity is more important than changes in the litter quality. There was some degree of correspondence between the treatment response of aboveground biomass production and that of nutrient turnover and decomposition (Figs. 1, 2), while differences in decomposition of litter among plant species was small (Fig. 3). The latter indicates that herbivory-induced changes in plant species composition, which has been suggested as a driver for herbivore-mediated changes of N dynamics^15,16,35^, had little effect on N dynamics in our community. Such herbivory-induced effects on N dynamics likely require large initial differences in plant litter quality, i.e. that there is a sharper contrast in litter quality between the plants that are preferred and those that are less preferred by the herbivores than we observed.

Since root feeding herbivores may influence performance of aboveground herbivores, and vice versa, by changes in plant quality^36,37^, we cannot rule out the possibility that the interactive effects are due to plant mediated interactions between above- and belowground herbivores. However, given the large effect on decomposition potential and N mineralization rate that we observed, we find it more likely that changes in total litter input, caused by altered biomass production and associated effects on the soil microbial community^38^, are driving the observed patterns. If correct, this is in accordance with the mechanism suggested to drive the feedbacks that large herbivores have on plant-soil feedbacks^5,39,40^.

## Conclusion

We demonstrate that N addition effects on an ecosystem can be subject to strong trophic control from spatially separated consumers. In our study, complex interactions with above- and belowground herbivores modulated the effect of N addition. Our results make evident the context-dependency of herbivory impacts on ecosystem functioning. Such context dependency has been demonstrated earlier, e.g. effects from insect herbivory depends on interaction with higher trophic levels^35^, or that herbivory effects depends on soil nutrient availability^20^. However, here we demonstrates that this influence differs also depending on whether herbivory occurs above or below ground, and that that the impact of insect herbivory on nutrient dynamics shifts dramatically due to N addition. Although we cannot provide a mechanistic explanation to the complex interactions we observed, our results highlight the importance of taking above- and belowground trophic interactions into consideration when predicting, modelling, or interpreting the ecological consequences of global change.

## Supplementary information

Supplementary Tables.
