# Supplementary material for: Below-ground herbivory mitigates biomass loss from above-ground herbivory of nitrogen fertilized plants
Source: Sci Rep. 2020 Jul 29;10:12752. doi: 10.1038/s41598-020-69696-3 (PMC7391681; doi:10.1038/s41598-020-69696-3)
Supplement: Supplementary file 1 — Supplementary Tables. [file 41598_2020_69696_MOESM1_ESM.pdf]

Electronic Supplemental Material: **Below-ground herbivory mitigates biomass loss from above-ground herbivory of nitrogen fertilized plants** by Pernilla Borgström, Riccardo Bommarco, Maria Viketoft, Joachim Strengbom.

Electronic Supplemental Information (ESM)

S1. The schematic figure shows the dimensions of the mesocosm set-up, and the randomized assignment of treatments within the experiment. Each block of eight mesocosms included one replicate of each of the eight treatment combinations, yielding 64 mesocosms in total.

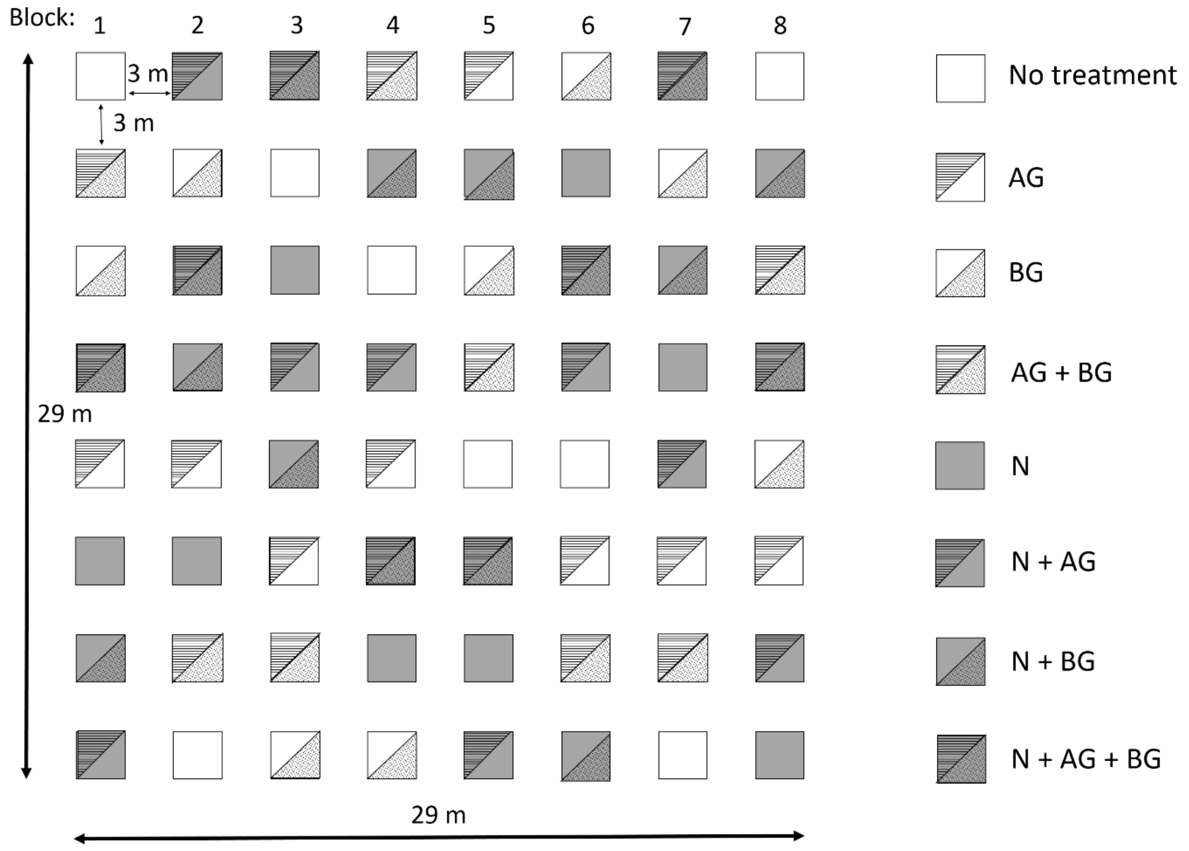

Electronic Supplemental Material: **Below-ground herbivory mitigates biomass loss from above-ground herbivory of nitrogen fertilized plants** by Pernilla Borgström, Riccardo Bommarco, Maria Viketoft, Joachim Strengbom.

S2. Treatment effects of nitrogen (N), aboveground herbivory (A) and belowground herbivory (B) on shoot biomass (aboveground biomass) in the final year (2015) of the experiment. Mass loss of read tea (red tea) was used as co-variates in the analysis. Bold type denotes statistical significance at p<0.05.

Treatment	F	P
Red tea	3.3	0.09
A	3.0	0.09
B	0.0	0.99
N	**6.8**	**0.01**
AxB	3.2	0.08
NxA	1.0	0.03
NxB	2.9	0.1
NxAxB	**4.5**	**0.04**

Electronic Supplemental Material: **Below-ground herbivory mitigates biomass loss from above-ground herbivory of nitrogen fertilized plants** by Pernilla Borgström, Riccardo Bommarco, Maria Viketoft, Joachim Strengbom.

S 3. Treatment effects of nitrogen (N), aboveground herbivory (A) and belowground herbivory (B) on shoot biomass (aboveground biomass) in the final year (2015) of the experiment. Nitrate production (N min) was used as co-variates in the analysis. Bold type denotes statistical significance at $p<0.05$.

Treatment	F	P
N min	2.9	0.09
A	2.6	0.12
B	0.01	0.90
N	**5.9**	**0.02**
AxB	2.8	0.1
NxA	1.2	0.30
NxB	2.3	0.1
NxAxB	**4.4**	**0.04**
